# A Case of Intestinal Granular Calcifications Associated With Phosphate Binder Use in a Patient on Hemodialysis

**DOI:** 10.1002/ccr3.70607

**Published:** 2025-07-07

**Authors:** Takaaki Nagao, Yosuke Azuma, Ryo Ichibayashi

**Affiliations:** ^1^ Department of Neurosurgery Toho University Sakura Medical Center Chiba Japan; ^2^ Division of Emergency Medicine Department of Internal Medicine Toho University Sakura Medical Center Chiba Japan

**Keywords:** calcification, hyperphosphatemia, lanthanum carbonate, radiographic findings, X‐ray

## Abstract

An experienced physician correctly identified a high‐intensity intestinal X‐ray from Lanthanum Carbonate, preventing unnecessary tests and enabling proper treatment. Awareness of this is vital in managing renal failure patients.

## 1 Clinical Picture

**FIGURE 1:**
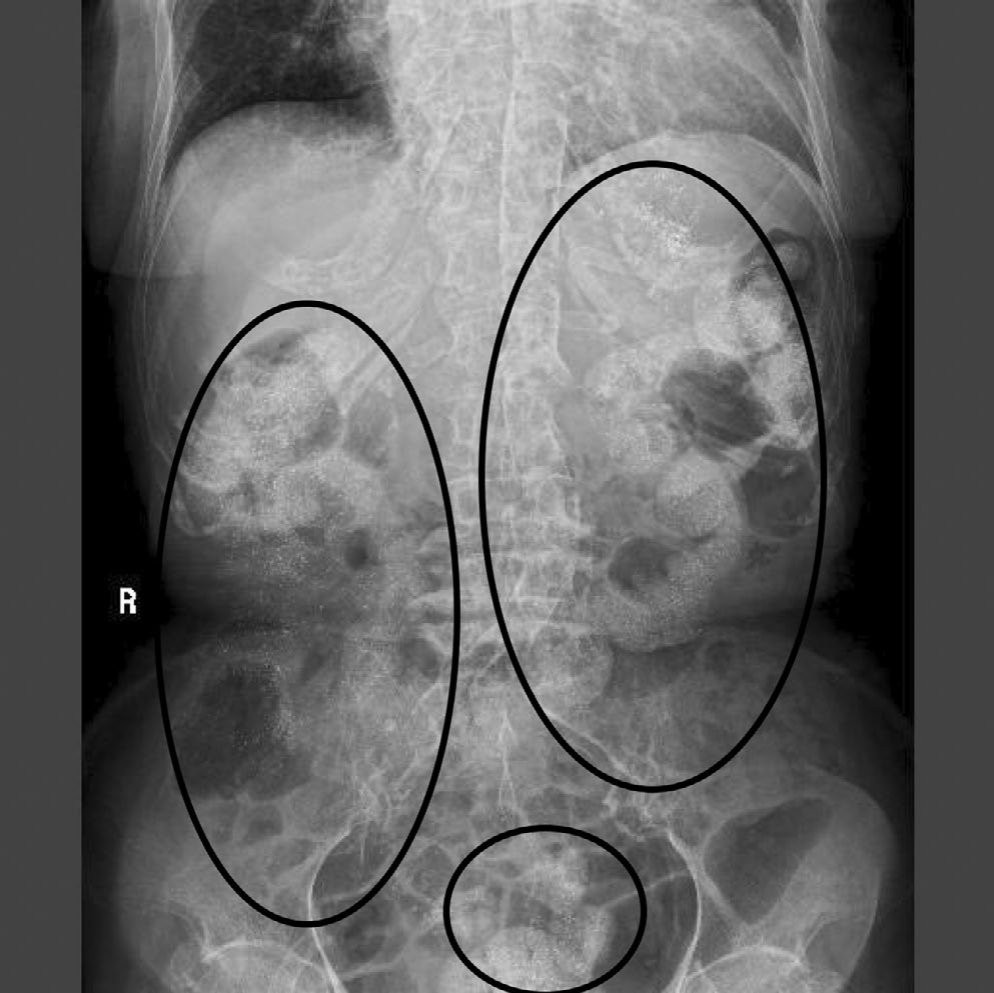
A vertebral fracture is observed in the 4^th^ lumbar vertebra. Fine, high‐intensity granular shadows are scattered throughout the area, consistent with intestinal gas (black circle).

An 80‐year‐old woman was undergoing hemodialysis three times a week and taking lanthanum carbonate hydrate granules (LCHG) for end‐stage renal failure due to diabetes. Two days before the visit, she had fallen at home and bruised her lower back. She had persistent lower back pain and difficulty walking, so she called an ambulance. On arrival, her vital signs were normal, and she had spontaneous pain and tenderness just above the lumbar vertebrae but no sensory or motor disturbances in the lower limbs. A dialysis shunt was confirmed in the left forearm. We performed a lumbar X‐ray and found a deformity in the fourth lumbar vertebra. A subsequent short inversion recovery (STIR) lumbar MRI showed a high signal in the fourth vertebra, and we diagnosed the fourth lumbar vertebra as having an acute compression fracture. However, scattered calcified components were found in the intestine during the X‐ray (Figure 1). The resident who provided the initial care to the emergency patient and the neurosurgeon seconded to the emergency department to study emergency care questioned the findings. Still, an experienced emergency physician pointed out that the oral administration of LCHG was the cause. Lanthanum carbonate is used to treat hyperphosphatemia in patients with renal failure. It adsorbs phosphate in the intestine and is excreted with the stool. Due to its chemical properties, it is known to be observed as a radiopaque on X‐ray images [1]. Although the radiopacity in the intestine caused by lanthanum carbonate is a known phenomenon, in clinical practice, inexperienced medical personnel may mistake it for an abnormal finding [2]. Lanthanum carbonate combines with phosphate in the intestine to form insoluble lanthanum phosphate. This combination is depicted as radiopaque. Furthermore, the size and distribution of the particles vary depending on the dosage form, intestinal contents, and peristalsis state, so it is often observed as a granular or dot‐like radiopaque. Even if lanthanum carbonate is taken orally, the radiopaque image may not be observed depending on the timing of the scan. Calcium‐ and iron‐based phosphate binders may appear as radiopaque images on X‐rays. On the other hand, polymer‐based phosphate binders are radiolucent and cannot be seen on images. Thus, understanding the characteristics of phosphate binders can streamline the emergency diagnostic process in patients with renal failure. In this case, the emergency physician promptly recognized the radiopaque intestinal findings as lanthanum‐related, avoiding unnecessary additional testing. This not only reduced the patient's burden but also promoted the efficient use of medical resources. Such knowledge is particularly valuable when a patient's medical history is unknown. This case highlights the potential for lanthanum‐induced intestinal radiopacity to be misinterpreted by inexperienced clinicians, emphasizing its practical relevance in preventing unnecessary investigations or misdiagnosis. However, as this is a single case report, the radiographic findings may vary among individuals. Caution is therefore warranted when interpreting similar images in clinical practice.

## Author Contributions

**Takaaki Nagao:** conceptualization, writing – original draft, writing – review and editing. **Yosuke Azuma:** visualization, writing – review and editing. **Ryo Ichibayashi:** project administration, supervision, writing – review and editing.

## Disclosure

Permission to Reproduce Material From Other Sources: None.

Clinical Trial Registration: Therefore, this case report does not involve a clinical trial and was not registered in a clinical trial registry.

## Ethics Statement

Ethical approval was not required for this case report, as all patient information was fully anonymized, and no identifiable data were included. Written informed consent was obtained from the patient for publication.

## Consent

Written informed consent was obtained from the patient to publish this report by the journal's patient consent policy.

## Conflicts of Interest

The authors declare no conflicts of interest.

## Data Availability

The data presented in this study are available on request from the corresponding author. The data are not publicly available due to privacy and ethical considerations.
